# Protocol: a systematic review of studies developing and/or evaluating search strategies to identify prognosis studies

**DOI:** 10.1186/s13643-017-0482-y

**Published:** 2017-04-20

**Authors:** Nadia Corp, Joanne L. Jordan, Jill A. Hayden, Emma Irvin, Robin Parker, Andrea Smith, Danielle A. van der Windt

**Affiliations:** 1 Arthritis Research UK Primary Care Centre, Research Institute of Primary Care and Health Sciences, Keele University, Keele, Staffordshire ST5 5BG UK; 2 Department of Community Health & Epidemiology, Dalhousie University, 5790 University Avenue, Room 403, Halifax, NS B3H 1V7 Canada; 3 Institute for Work & Health, 481 University Avenue, Suite 800, Toronto, ON M5G 2E9 Canada; 4 W.K. Kellogg Health Sciences Library, Dalhousie University, 5850 College St, Halifax, NS B3H 4R2 Canada; 5 Dalhousie University, Halifax, NS Canada

**Keywords:** Prognosis, Search filter, Performance, Systematic review

## Abstract

**Background:**

Prognosis research is on the rise, its importance recognised because chronic health conditions and diseases are increasingly common and costly. Prognosis systematic reviews are needed to collate and synthesise these research findings, especially to help inform effective clinical decision-making and healthcare policy.

A detailed, comprehensive search strategy is central to any systematic review. However, within prognosis research, this is challenging due to poor reporting and inconsistent use of available indexing terms in electronic databases. Whilst many published search filters exist for finding clinical trials, this is not the case for prognosis studies.

This systematic review aims to identify and compare existing methodological filters developed and evaluated to identify prognosis studies of any of the three main types: overall prognosis, prognostic factors, and prognostic [risk prediction] models.

**Methods:**

Primary studies reporting the development and/or evaluation of methodological search filters to retrieve any type of prognosis study will be included in this systematic review. Multiple electronic bibliographic databases will be searched, grey literature will be sought from relevant organisations and websites, experts will be contacted, and citation tracking of key papers and reference list checking of all included papers will be undertaken. Titles will be screened by one person, and abstracts and full articles will be reviewed for inclusion independently by two reviewers. Data extraction and quality assessment will also be undertaken independently by two reviewers with disagreements resolved by discussion or by a third reviewer if necessary.

Filters’ characteristics and performance metrics reported in the included studies will be extracted and tabulated. To enable comparisons, filters will be grouped according to database, platform, type of prognosis study, and type of filter for which it was intended.

**Discussion:**

This systematic review will identify all existing validated prognosis search filters and synthesise evidence about their applicability and performance. These findings will identify if current filters provide a proficient means of searching electronic bibliographic databases or if further prognosis filters are needed and can feasibly be developed for systematic searches of prognosis studies.

**Electronic supplementary material:**

The online version of this article (doi:10.1186/s13643-017-0482-y) contains supplementary material, which is available to authorized users.

## Background

Prognosis has been recognised as an important area of research especially given the growing number of people worldwide living longer with one or more chronic health condition or disease [1]. Studies of prognosis provide information on the likely future course of a disease or health condition (overall prognosis studies), prognostic factors associated with future health outcomes (prognostic factor studies), and patients’ individual risk of future outcome based on multiple prognostic factors (prognostic model studies) [1–3]. Prognosis research is important for patients, clinicians, health care providers and health researchers to inform effective clinical decision-making, healthcare policy regarding diagnosis, therapeutic interventions and health care service delivery, and the planning of future studies. Furthermore, results from prognosis research are increasingly used in the rapidly emerging field of stratified or precision medicine [4], where treatments are targeted to those patients most likely to respond (or experience least harm), requiring evidence regarding prognostic factors and/or predictors of differential treatment response [5]. As the literature on prognosis increases, so too has interest in prognostic systematic reviews to collate and synthesise the research findings.

A detailed search strategy, at the core of all systematic reviews, aims to identify as many eligible studies as possible, whilst minimising the return of non-eligible studies, i.e. increasing precision. Such strategies remain problematic with regards to prognosis due to inconsistent and poor reporting of methods within the title and abstract of articles, a lack of specific indexing terms, and inconsistent use of indexing terms that are available. Methodological search filters are pre-tested search strategies for locating relevant literature within a given database. Whilst many search filters have been developed and evaluated for identifying studies and reviews of therapeutic interventions in online databases [6], this is not the case for prognosis studies. The Cochrane Methods Prognosis group has identified prognosis search strategies as a methodological priority for the development of this type of systematic review [7].

Two recent studies have addressed prognosis search filters. Chatterley and Dennett [8] examined the use of prognosis search filters in systematic reviews, concluding only a small proportion (5%) use a validated filter and that none of the published filters were highly sensitive or precise. A second study by Geersing et al. [9] validated and updated two search filters for prediction studies in Medical Literature Analysis and Retrieval System Online (MEDLINE) and found viable filters to identify prognostic [risk prediction] model studies but none that identified other types of prognosis studies (such as prognostic factor studies or studies on the impact of using prognostic [risk prediction] models). However, neither study conducted or identified a systematic review with a comprehensive search to identify the search filters; thus, potentially relevant filters with improved metrics may have been missed.

This systematic review aims to find and compare existing methodological filters that are developed and evaluated to identify any type of prognosis study. The findings will help elucidate current prognosis filter performance and inform the future development of a new, improved filter for use in prognosis systematic reviews.

## Methods

The objectives of this systematic review are as follows:

1. Identify search filters that aim to retrieve any type of prognosis studies (overall prognosis, prognostic factors, prognostic [risk prediction] models) that are published and/or freely available on the internet;
2. Identify the corresponding studies that evaluate these search filters;
3. Assess the methodological quality of the evaluation studies;
4. Compare content of the search strategies to identify commonly used terms;
5. Draw conclusions on the need and feasibility of developing a new prognosis filter.

Where applicable, the PRISMA-P checklist was used to develop this protocol [10] (see Additional file 1 for completed checklist). This systematic review was not registered on PROSPERO (the International Prospective Register of Systematic Reviews) as reviews concerning methodological issues alone and lacking a direct patient or clinical outcome do not currently meet the inclusion criteria for the register [11].

### Search methods

The following sources will be searched to identify search filters for prognosis studies and studies that developed and/or evaluated these filters:

- Electronic bibliographic databases: MEDLINE (Ovid), Excerpta Medica Database (EMBASE) (Ovid), Cumulative Index to Nursing and Allied Health Literature-Plus (CINAHL-P) (EBSCO), Web of Science (Science Citation Index Expanded (SCI-EXPANDED), Conference Proceeding Citation Index-Science (CPCI-S)), Library, Information Science & Technology Abstracts (LISTA; EBSCO), Library and Information Science Abstracts (LISA; ProQuest), and the Cochrane Library, these will be searched from inception up to the date the search is conducted;
- Publications reporting on methods from organisations producing evidence-based clinical guidelines, evidence syntheses and health technology assessments, including Scottish Intercollegiate Guidelines Network (SIGN), National Institute for Health and Care Excellence (NICE), Scientific Resource Center Methods Library of the Agency for Healthcare Research and Quality (AHRQ) Effective Health Care Program, McMaster’s University Health Information Research Unit, Canadian Agency for Drugs and Technologies in Health (CADTH) and British Medical Journal (BMJ) Clinical Evidence methods;
- Relevant web-based databases and repositories, such as OpenGrey, InterTASC, Meth4ReSyn citeulike library, other citeulike and mendeley groups, and the Cochrane Methodology Register;
- Conference abstracts from systematic review and library sciences methods conferences: Cochrane Colloquium; Medical Library Association (USA); European Association for Health Information and Libraries (EAHIL); Health Libraries Group (UK); Evidence Based Library and Information (International); Canadian Health Libraries Association (Canada);
- General search engines (first 10 pages of results), Google and DuckDuckgo;
- Hand-search of relevant journals, including Evidence Based Library and Information Practice (EBLIP), Health Information and Libraries Journal, Research Synthesis Methods, Systematic Reviews and Journal of the Canadian Health Libraries Association (JCHLA).

The search strategies will include terms for prognosis and search filters, utilising text word searching in key fields, including the title and abstract, combined with the databases’ Subject Headings (e.g. MeSH). See Additional file 2 for a draft of the MEDLINE search strategy, which will be adapted to the search capabilities of the other databases and platforms searched.

To identify further relevant search filters or publications about such filters, we will also contact researchers who specialise in information retrieval and have published widely in this field, undertake citation tracking of key papers and check the reference lists from all articles included in this review.

### Eligibility criteria

#### Inclusion criteria

Primary studies of any design concerned with the development and/or evaluation of methodological search filters to retrieve any type of prognosis studies, including the following:

- Development studies: studies in which a new filter is conceived, tested in a reference set of prognosis studies and the performance reported;
- Evaluation studies: studies in which a filter is tested in a new reference set and performance reported. Whilst ideally the filter will have a corresponding development study published, we anticipate that this will not always be the case;
- Development and evaluation studies of a newly designed filter tested against the performance of an existing filter that may have previously been published;
- Comparative studies where the performance of a number of filters are compared against each other.

There will be no restrictions on language or publication period.

#### Exclusion criteria

Prognosis studies which do not specifically develop or test a prognosis search filter will be excluded. Conference abstracts will be excluded if all the necessary data is not available or cannot be obtained from the author. Search filters where no corresponding development and/or evaluation study can be found will also be excluded from the review. However, these search strategies will be included in a comprehensive list of filters for future reference.

### Study selection

All references identified will be downloaded and imported from databases and journals or entered manually into Endnote and duplicates removed. To assist in managing screening, quality assessment and data extraction across continents; unique references will then be transferred into Covidence computer software [12].

One reviewer will exclude articles clearly irrelevant from the title. Titles and abstracts of the remaining articles will be independently assessed by two reviewers against the eligibility criteria, and articles will be excluded by agreement. Disagreements will be resolved by discussion or referral to a third reviewer. If it is unclear as to whether the publication is relevant or not, it will be included for the next step. Full-text copies of all the remaining articles will be obtained and assessed against the eligibility criteria by two independent reviewers in the same way as the abstract screening, with a third reviewer resolving any disagreements, if necessary. The reasons for excluding each paper at the full-text stage will be recorded.

### Data extraction

Two reviewers will independently extract data using a pre-designed data extraction form, which will be piloted and refined in advance of full data extraction. The following data will be extracted:

- Article characteristics e.g. authors, publication year, study ID;
- Type of filter study (i.e. development, evaluation, or both);
- Filter study characteristics e.g. search dates, databases used, clinical area of study and number of filters developed or evaluated;
- Details of each filter developed and/or evaluated e.g. type of filter, terms used and performance measures;
- Reference set characteristics e.g. method of identification of reference set, inclusion of gold and non-gold standard records, inclusion criteria and size;
- Presence of internal and external validity testing;
- Study limitations;
- Comparison of filter performance against other published filters;
- Assessment of whether search strategy is described in sufficient detail to be reproduced.

Where a study reports on multiple filters, results in terms of measures of performance will be extracted for each filter. In the case of development studies where data is presented for the sensitivity and precision of individual search terms, only single-term filters that the original authors selected as reporting the best performance will be extracted, while all multiple-term filters will be extracted.

Authors will be contacted for missing information necessary for data synthesis.

### Quality assessment

All articles meeting the inclusion criteria will be independently assessed for quality by two reviewers, with any disagreements resolved though discussion or referral to a third reviewer. Quality assessment will be informed by several previously developed search filter appraisal tools: UK InterTASC Information Specialists’ Sub-Group (ISSG) Search Filter Appraisal Checklist [13], the Canadian Agency for Drugs and Technologies in Health Critical Appraisal (CADTH CAI) and Ranking Tool for Search Filters [14], and Jenkin’s search filter appraisal checklist [15], as well as Benyon et al.’s [16] Cochrane review that assessed filter development and evaluation studies for diagnostic test accuracy. Unique items from these tools have been amalgamated into a review-specific quality assessment checklist (see Additional file 3) that will be piloted with all reviewers alongside the data extraction form before the beginning this stage of the review.

### Data synthesis

The PRISMA statement guidelines will be followed when reporting the findings of this systematic review [17].

The filters identified will be summarised and their characteristics reported. Filters will be grouped by the database and platform for which they were developed, and also by type of prognosis study, i.e. overall prognosis, prognostic factors and prognostic [risk prediction] models. These groups will facilitate comparison of the specific terms used.

We will tabulate performance metrics reported in development and evaluation studies, i.e. specificity, specificity, precision, accuracy and number needed to read according to filter; this will allow comparisons between the performances reported in the original development (and evaluation) study and that of subsequent evaluation studies. If a specific metric is not reported, it will be calculated from the available information, where possible [16].

For each of the three types of prognosis study, filters will be sub-grouped according to aim of the filter with respect to sensitivity-maximising, precision-maximising, specificity-maximising or balance of sensitivity and specificity/precision, and performance metrics compared.

Limitations of the search filter studies will be noted, as highlighted in the quality assessment, including whether the filter performance can be generalised to other subject areas. Possible categorization of the limitations include—but are not limited to—methods of creating reference sets, performance specific to a clinical topic, and other aspects of the methods to evaluate or develop the search filter. Performance metrics will be examined with reference to these potential limitations.

In addition, an inventory of existing methodological search filters for prognostic studies, regardless of whether an evaluation and/or development paper has been published, will be compiled for future reference.

## Discussion

To date, there has been no systematic review that has specifically assessed the current status of methodological filters for identifying any type of prognosis studies (overall prognosis, prognostic factors, prognostic [risk prediction] models). This systematic review aims to address this omission and to assess the applicability and performance of identified filters. These findings will enable us to identify whether any existing filters provide a proficient means of searching electronic bibliographic databases or if further prognosis filters need developing and whether they can feasibly be developed for systematic searches of prognosis studies.

## Additional files

Additional file 1: PRISMA-P Checklist. (DOCX 37 kb)

Additional file 2: Draft search strategy for MEDLINE (Ovid). (DOCX 17 kb)

Additional file 3: Quality appraisal tool. (DOCX 19 kb)

## Abbreviations

AHRQ: Agency for Healthcare Research and Quality
BMJ: British Medical Journal
CADTH: Canadian Agency for Drugs and Technologies in Health
CADTH CAI: Canadian Agency for Drugs and Technologies in Health Critical Appraisal
CINAHL-P: Cumulative Index to Nursing and Allied Health Literature - Plus
CPCI-S: Conference Proceeding Citation Index – Science
EAHIL: European Association for Health Information and Libraries
EBLIP: Evidence Based Library and Information Practice
EMBASE: Excerpta Medica Database
ISSG: Information Specialists' Sub-Group
JCHLA: Journal of the Canadian Health Libraries Association
LISA: Library and Information Science Abstracts
LISTA: Library, Information Science & Technology Abstracts
MEDLINE: Medical Literature Analysis and Retrieval System Online
MeSH: Medical Subject Headings
NICE: National Institute for Health and Care Excellence
PRISMA: Preferred Reporting Items for Systematic Review and Meta-Analysis
PRISMA-P: Preferred Reporting Items for Systematic Review and Meta-Analysis-Protocols
PROSPERO: International Prospective Register of Systematic Reviews
SCI-EXPANDED: Science Citation Index Expanded
SIGN: Scottish Intercollegiate Guidelines Network
