# Supplementary material for: Protocol: a systematic review of studies developing and/or evaluating search strategies to identify prognosis studies
Source: Syst Rev. 2017 Apr 20;6:88. doi: 10.1186/s13643-017-0482-y (PMC5399431; doi:10.1186/s13643-017-0482-y)
Supplement: Supplementary file 2 — Draft search strategy for MEDLINE (Ovid). (DOCX 17 kb) [file 13643_2017_482_MOESM2_ESM.docx]

**Additional file 2**

**Draft search strategy for MEDLINE (Ovid)**

|  | **Searches** |  |
| --- | --- | --- |
| 1 | "Information Storage and Retrieval"/ |  |
| 2 | ((information or literature or study or studies) adj3 retriev$).mp. |  |
| 3 | (search$ adj3 (strateg$ or filter$ or hedge$ or technique$ or term$1)).mp. |  |
| 4 | (retriev$ adj3 (strateg$ or filter$ or hedge$ or technique$ or term$1)).mp. |  |
| 5 | (search$ adj3 (precision or recall or accura$ or sensitiv$ or specific$ or efficiency or comprehensiveness)).mp. |  |
| 6 | (retriev$ adj3 (precision or recall or accura$ or sensitiv$ or specific$ or efficiency or comprehensiveness)).mp. |  |
| 7 | ((methodology or methodologic$) adj3 (strateg$ or filter$ or hedge$ or technique$ or term$1)).mp. |  |
| 8 | or/1-7 |  |
| 9 | exp disease progression/ |  |
| 10 | prognos$.mp. |  |
| 11 | (prediction$ or predictor$).mp. |  |
| 12 | progression.mp. |  |
| 13 | (disease adj3 course).mp. |  |
| 14 | modifier$1.mp. |  |
| 15 | mediator$1.mp. |  |
| 16 | natural histor$.tw. |  |
| 17 | ((precision or individuali?ed or personali?ed or stratified or systems) adj medicine).mp. |  |
| 18 | exp models, statistical/ |  |
| 19 | cohort$.mp. |  |
| 20 | (follow-up$ adj3 stud$).mp. |  |
| 21 | ("case control" or "case controlled").mp. |  |
| 22 | (prospective$ adj3 stud$).mp. |  |
| 23 | (longitudinal$ adj3 stud$).mp. |  |
| 24 | (observation$ adj3 stud$).mp. |  |
| 25 | "time series".mp. |  |
| 26 | or/9-25 |  |
| 27 | 8 and 26 |  |
| 28 | (prognos$ adj5 (strateg$ or filter$ or hedge$)).mp. |  |
| 29 | (prediction adj5 (strateg$ or filter$ or hedge$)).mp. |  |
| 30 | or/27-29 |  |
