# Supplementary material for: Protocol: a systematic review of studies developing and/or evaluating search strategies to identify prognosis studies
Source: Syst Rev. 2017 Apr 20;6:88. doi: 10.1186/s13643-017-0482-y (PMC5399431; doi:10.1186/s13643-017-0482-y)
Supplement: Supplementary file 3 — Quality appraisal tool. (DOCX 19 kb) [file 13643_2017_482_MOESM3_ESM.docx]

**Additional file 3**

**Draft quality appraisal of filter**

We have designed a quality appraisal tool, based on several previously developed search filter appraisal tools: UK InterTASC Information Specialists' Sub-Group (ISSG) Search Filter Appraisal Checklist [10], the Canadian Agency for Drugs and Technologies in Health Critical Appraisal (CADTH CAI) and Ranking Tool for Search Filters [11] and Jenkin’s search filter appraisal checklist [12], as well as consideration of Benyon *et al.*’s (2013)[13] Cochrane review which assessed filter development and evaluation studies for diagnostic test accuracy.

Please answer each statement Yes, No or Unclear, unless otherwise stated. Note: a Yes response is indicative of good quality.

|  |  | | | **Yes/No/Unclear** |
| --- | --- | --- | --- | --- |
| **Intended application of filter** | | | |  |
| **1** | Is the purpose of the filter clearly stated *e.g.* precision/sensitivity, database/interface? | | |  |
| **2** | Is the intended audience clearly stated *e.g.* research, clinician? | | |  |
| **Filter design** | | | |  |
| **3** | Is the derivation of search terms clearly described? | | |  |
| **4** | Are the terms used and the way they are combined likely to retrieve all studies of interest? | | |  |
| **Gold standard** | | | |  |
| **5** | Was a gold standard used? If No, go to ‘Filter validation’ | | |  |
| **6** | Are there limitations to the gold standard(s)? if so which (tick) | | |  |
| **6a.** | | Not developed in an appropriate manner (e.g. a priori inclusion/exclusion criteria, appropriate search method, absence of prognosis search strategy) |  |  |
| **6b.** | | Not generalizable to the whole body of the relevant literature in terms of the topic, years and types of journals covered? |  |  |
| **6c.** | | No reasonable justification for the sample size of the gold standard? |  |  |
| **6d.** | | Other, please state……………………………………………………………………………. | |  |
| **Filter validation** | | | |  |
| **7** | Was the filter validated on a gold standard set? If No, go to ‘Applicability’ | | |  |
| **8** | Was the filter was tested on a different set than the gold standard set it was derived from | | |  |
| **9** | The performance measures of the filter on the validation gold standard is clearly reported e.g. precision, specificity, sensitivity | | |  |
| **Applicability/generalizability** | | | |  |
| **10** | Is the filter translatable to other interfaces/platforms for the same database? | | |  |
| **11** | Has the filter been tested further, by the same or different authors, in different contexts *e.g.* settings, topicsc? | | |  |
| **12** | Are there other potential limitations to this research? | | |  |
